# Effects of Mobile-Based Rehabilitation in Adolescent Football Players with Recurrent Lateral Ankle Sprains during the COVID-19 Pandemic

**DOI:** 10.3390/healthcare10030412

**Published:** 2022-02-22

**Authors:** Xiaobo Qu, Kai Li, Sangcheul Nam

**Affiliations:** 1 College of Physical Education, Pingdingshan University, Pingdingshan 467000, China; 3053@pdsu.edu.cn; 2 Department of Physical Education, Jeonbuk National University, Jeonju 54896, Korea; 3050@pdsu.edu.cn

**Keywords:** lateral ankle sprain, football, supervised rehabilitation, mobile-based rehabilitation, dynamic balance

## Abstract

Football is a sport involving dynamic movements, and ankle sprains are common sports injuries experienced by football players. Ankle sprains exhibit a high recurrence rate, and rehabilitation training is effective; however, expert-supervised rehabilitation (SVR) at training centers is difficult due to the recent COVID-19 pandemic. This study investigated the effects of mobile-based rehabilitation (MBR) performed at home by high school football players. Sixty players (SVR: 30 and MBR: 30) with recurrent ankle sprains were analyzed. The rehabilitation program consisted of strength and balance training, and the training intensity was gradually increased from week 1 to week 8. The SVR group underwent training at the center with experts, and the BMR group were provided with programs and feedback using mobile devices. Ankle muscle strength was evaluated by measuring isometric eversion, inversion, plantarflexion, and dorsiflexion contraction using a hand-held dynamometer, and dynamic balance was assessed using the Y-balance test (YBT; anterior, posteromedial, and posterolateral); the Foot and Ankle Outcome Score (FAOS) was used for the subjective evaluation. Measurements were conducted at weeks 1, 4, and 8. The patients visited the clinic within 1 week after the injury, and the first test was conducted after consent to participate in the research. Patients underwent the second test at an average of 3.2 weeks after the first test, and the last test at an average of 4.4 weeks after the second test. Although only the SVR group exhibited improvement in strength (eversion and dorsiflexion), YBT and subjective satisfaction at week 4, these measurements improved in both the SVR and MBR groups at week 8. Therefore, mobile–based rehabilitation could be a suitable alternative for high school athletes with ankle sprains who cannot undergo supervised rehabilitation.

## 1. Introduction

Football is a popular sport globally, with active participation by many countries and individuals. In football players, due to the frequent physical contact and dynamic movements, ankle sprains are the most common sports injuries [1,2,3]. 

Ankle sprains occur as a traumatic event, and high-energy external loads cause ankle inversion and supination when landing or when a rapid center of gravity shift in an unstable foot posture occurs. If the external load is so great at this moment that control of the foot is lost, the anterior talofibular ligament or calcaneofeluar ligament is damaged [4,5].

The mechanism of injury in lateral ankle sprains is usually caused by ankle inversion and supination on landing, and the anterior talofibular ligament or calcaneofeluar ligament on the lateral side is often injured The primary characteristic of ankle sprains is that the initial injury is acute, but it can also become chronic [6]. The recurrence rate of ankle sprains is 12–47%, and 20–32% of patients with sprains progress to chronic ankle instability [7,8]. Moreover, mechanical instability, such as ligament laxity, is a typical intrinsic cause and appears to occur frequently during adolescence [8,9]. Non-surgical treatments, including braces, fixation, and exercise rehabilitation for lateral ankle sprains are reported to provide positive effects [10]. According to a study by Vuurberg et al. [11], an ankle sprain prevention program reduced the risk of recurrence by 38%.

The rehabilitation program mainly comprises ankle strengthening, ankle balance, and coordination training, and the effect of the program can last up to 12 months [11,12]. However, only 6.8% of patients received physical therapy within 30 days of ankle injury, only 6.2% received ankle strengthening exercises, and 1.9% received balance training [10]. This means that the treatment of ankle sprains is not adequately performed. In another study, many high school athletes report returning to sports within a week of sprained ankles, and these findings show that they still do not receive systematic management [13].

One of the reasons is that academically active high school students often do not have easy access to specialized training centers for rehabilitation on a regular basis. Moreover, training at rehabilitation centers or facilities is currently limited owing to the COVID-19 pandemic [14,15]. Therefore, if a mobile-based rehabilitation (MBR) program that can be implemented at home is provided, participation in rehabilitation may be encouraged [16].

In previous home-based training study, it was reported that 8 weeks of proprioception training was performed in patients with ankle sprains, and resulted in a 35% reduction in recurrence in the following year [17]. Meanwhile, in another study, center-based training was recommended over home-based training for athletes in competitive sports [18]. So, while home-based training is generally helpful, it is still controversial compared to center-based training, and further research is needed.

Therefore, this study was conducted to compare the effectiveness of supervised rehabilitation (SVR) by experts at a training center and MBR programs performed at home by football players with recurrent ankle sprains. Although the program was only 8 weeks, an interim test and final measurement were performed to confirm the short-term change pattern. This study hypothesized that the MBR training program would produce positive results in regard to muscle strength, dynamic balance, and subjective satisfaction.

## 2. Materials and Methods

### 2.1. Participants

High school football players (age: 15–18 years, *n* = 103) who experienced ankle sprains and volunteered to participate after being informed through bulletin boards and research guide materials were enrolled in this study. Orthopedic surgeons selected Grade I and II athletes who had experienced recurrent ankle sprains and did not require surgery. For the purpose of this study, only athletes who had suffered repeated injuries, that is, twice or more within the previous month were included, and the tests and training were initiated within 7 days of the injury [19]. The study was conducted in accordance with the guidelines of the Declaration of Helsinki and approved by the Institutional Review Board of Pingdingshan University (PDSUIRB 20211020, 5 May 2021). All participants individually provided written informed consent, and consent was provided by the coaching staff or legal guardians.

**Figure 1:**
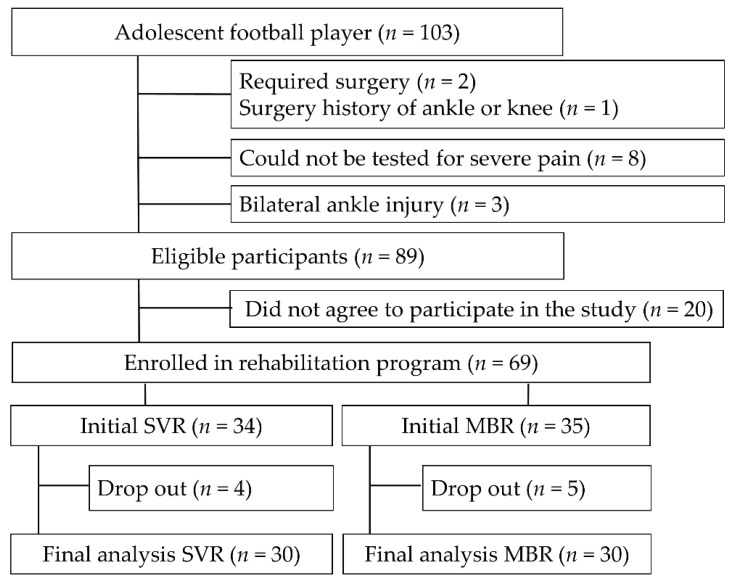
Schema illustrating study flow.

After initiation of the program, participants were excluded from the final analysis due to contact loss, discontinuation due to improvement, and choice of oriental medicine and other treatment methods (*n* = 9). Therefore, the final analysis involved 60 players (SVR: 30; MBR: 30). SVR or MBR training was selected by the players (Figure 1).

In the SVR group, if several players were members of one team, the researcher conducted visiting training. The athletes visited if they resided in close proximity to the training center. The MBR group was provided with the program to be followed; a booklet was made into a video for use on a mobile device, and the training was performed on an individual basis at home.

### 2.2. Rehabilitation Program

A previous study reported that the rehabilitation dropout was high, and only 23% completed an 8-week rehabilitation program [19]. Therefore, the rehabilitation program in this study was conducted for eight weeks (Table 1). The tests were performed at 1, 4, and 8 weeks. Patients underwent the second test at an average of 3.2 weeks after the first test, and the last test at an average of 4.4 weeks after the second test. 

**Figure 2:**
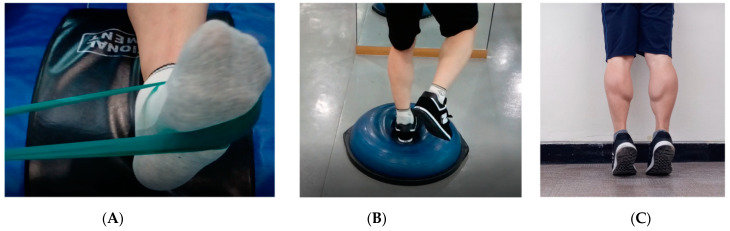
Photographs showing (**A**) ankle strength training with tube band; (**B**) balance training with BOSU; (**C**) heel raise strength training with body weight.

Rehabilitation training consisted of a 30-min daily strength and balance improvement program with reference to previous literature (Figure 2) [19,20,21]. To accurately match the quantity of training in the two groups, detailed information regarding the type of training, duration, frequency, intensity, and method were provided. The training period was 5 days per week for 8 weeks, and the intensity and duration of the training sessions were gradually increased every 2 weeks. The ratio of strength training to balance training was 50:50. Athletes maintained a training log, and researchers verified the amount of exercise in the two groups, encouraged continued participation, and provided feedback and counseling.

Ankle strengthening exercises included the use of a tube band (TheraBand, Hygenic Corp., Akron, OH, USA), with ankle and lower extremity strengthening using body weight. The tube training included ankle inversion, eversion, dorsiflexion, and plantar flexion. For lower extremity training, body weight, squats, heel raises, and toe-ups were performed. The intensity was gradually increased by altering the color of the band, and increasing the amount of exercise, number of sets, and frequency.

The balance training was performed using a BOSU ball (NexGen, Ashland, OH, USA). The difficulty level gradually increased from static to dynamic, two-legged stand to one-legged stand, stand stop to change direction, and two-legged squat to one-legged squat. The training time with the device was gradually increased. Safe training methods were taught to prevent falls, and any dangerous objects in the surrounding area were removed.

### 2.3. Ankle Strength Test

After completing the questionnaire, athletes performed a warm–up and stretching for approximately 20 min before the ankle strength and balance tests were conducted. Subsequently the isometric maximum inversion, eversion, plantarflexion, and dorsiflexion strengths were measured using a portable hand-held dynamometer (Power Track II Commander Muscle Tester, JTECH Medical, Midvale, UT, USA) [22]. This test is relatively simple and convenient, and can therefore be readily understood by patients. It has a relatively lower cost than the isokinetic muscle strength measurement, known as the gold standard, and has high reliability and validity [23,24]. The intra-class correlation coefficients of ankle inversion and extraversion were up to 0.815 and 0.829, respectively [22].

**Figure 3:**
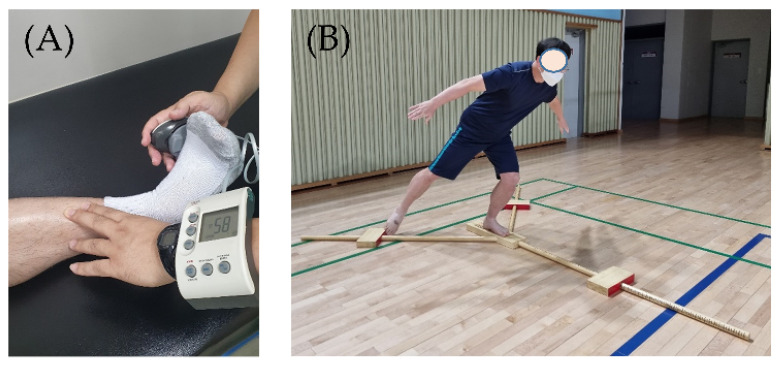
Ankle strength and dynamic balance test; (**A**) Hand-held dynamometer strength test; (**B**) Y-balance test.

The measurement method was carried out with reference to the prior literature [22,25]. The participant was in a supine position on the measuring table, and the pelvis and thigh were fixed with a belt so that the body did not move. During the measurement, the participant held the handle. The ankle posture was an anatomically neutral position, and the measurement angle was performed at 0 degrees. The examiner stood beside the patient’s tested foot. The direction of the foot was maintained in a neutral position, and the examiner instructed the participant to exert maximum muscle strength. One hand of the examiner stabilized the ankle just above the malleolus bone (Figure 3A). A dynamometer was placed on the lateral metatarsal head for the eversion testing. When the patient exerted muscle strength, the examiner provided resistance and measured the muscle strength isometrically. Inversion, plantarflexion, and dorsiflexion were performed in the same manner. The procedure was conducted several times with detailed explanations until the patient gained familiarity with the measuring equipment. The healthy ankle was examined first, followed by the injured ankle. The test was performed twice, and the higher value was recorded in kilograms.

### 2.4. Y-Balance Test

After the ankle muscle strength test, patients were provided with a rest period of 10–20 min for physiological recovery [26]. Dynamic balance was measured using the Y-balance test (YBT) (Figure 3B). The YBT was conducted using an inverted Y-shaped device, and the front was anterior, the posterior on both sides were 135° apart, and the posteromedial and posterolateral were spread in three directions [27]. The patient stood on one leg, and the distance was measured by extending the leg as far as possible. To increase the participants’ understanding and to gain familiarity, we practiced more than three times, and the examiner demonstrated the procedure with explanations.

The healthy leg of the participant was examined first. The participant removed their shoes and stood on one foot on a plate at the center. First, anterior, posteromedial, and posterolateral measurements were performed. If the patient kicked the measuring device or lost balance and both feet touched the ground, it was considered a foul, and the test was repeated after 5 min of rest. The experiment was conducted three times and the average value was used. The formula used was as follows:

Score = ((sum of distances in three directions)/(length of lower extremity × 3)) × 100

The leg length used in the formula is the distance from the anterior superior iliac spine to the center of the ipsilateral medial malleolus. A safe environment was established to exclude the risk of falls. In the event of a fall, an inspector monitored the area and an anti–skid environment was created to prevent collision.

### 2.5. Self-Evaluation Using Questionnaires

Two questionnaires were used for self-evaluation: the foot and ankle outcome score (FAOS) and identification of functional ankle instability questionnaire (IDFAI). The FAOS consists of 42 items in five categories: pain (9 items), symptoms (7 items), functions of daily living (17 items), functioning in sports and recreation (5 items), and foot/ankle-related quality of life (4 items) [28]. A score of 0 indicated the worst condition of the ankle, and 100 indicated the best condition. The IDFAI is a tool used to investigate ankle sprains and ankle instability. It comprises 10 items, and the frequency of sprains, “giving way,” and being “unstable” are determined. In this study, questions related to the sprain were extracted and analyzed [29,30]. For the subjective evaluation of participants, writing was done by hand, and when it was necessary to clarify the meaning of a statement, the researcher assisted.

### 2.6. Data Analysis

The sample size was calculated using G*power software (G*power 3.1, University of Düsseldorf, Düsseldorf, Germany): effect size, f = 0.25; α error = 0.05; power, (1−β err prob) = 0.99, two groups, and three measurements at the repeated measures, within-between interaction. The results were Critical F = 3.074; Denominator df = 116; Actual power = 0.99; Total sample size = 60. [31]. The data analysis was performed using SPSS Statistics software (version 25.0; IBM Corp., Armonk, NY, USA). The normality tests were performed using the Kolmogorov–Smirnov and Shapiro–Wilk tests. Parametric analyses were performed because the main variables analyzed were normally distributed. Data are expressed as means and standard deviations for continuous variables and as numbers and percentages for categorical variables. An independent *t*-test was performed for comparison between the groups, one-way repeated measures ANOVA was used for intragroup comparison, and Bonferroni correction was applied for the post hoc test [32]. Categorical variables, such as position, dominant side, and rate of injury, were analyzed using the chi-square test. A repeated two-way ANOVA was performed to observe group and time interactions. Statistical significance was set a priori at *p* < 0.05.

## 3. Results

### 3.1. General Characteristics of Participants

Table 2 presents the participants’ general characteristics. Comparisons were made between the SVR and MBR groups, and there were no significant differences in age (*p* = 0.390), height (*p* = 0.528), weight (*p* = 0.805), and body mass index (BMI) (*p* = 0.255). There were no significant differences between the groups regarding the playing position (*p* = 0.405) and injury side (*p* = 0.795).

### 3.2. Ankle Strength Test Using Hand Held Dynamometer

**Figure 4:**
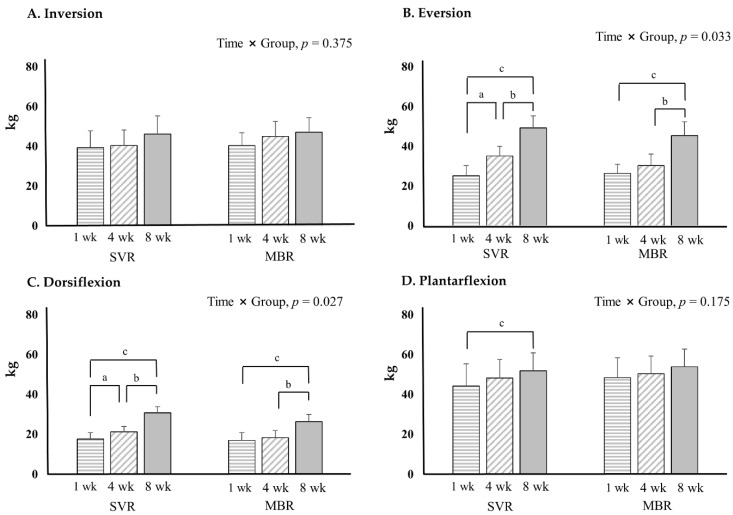
Ankle strength results with hand held dynamometer. *p* < 0.05; significance, a = 1 week versus 4 weeks; b = 4 weeks versus 8 weeks; c = 1 week versus 8 weeks. Abbreviations: SVR, supervised based rehabilitation; MBR, mobile-based rehabilitation, wk; week.

Eversion and dorsiflexion exhibited significant improvement in muscle strength at 8 weeks compared to those at 1 week in both the SVR and MBR group. More specifically, in the SVR group, at week 4, eversion and dorsiflexion were significantly improved compared to those at week 1. However, there was no significant change at week 4 in the MBR group. The improvement based on the time and group increased significantly in SVR compared to that in MBR (Figure 4).

### 3.3. Dynamic Balance with Y-Balance Test

**Figure 5:**
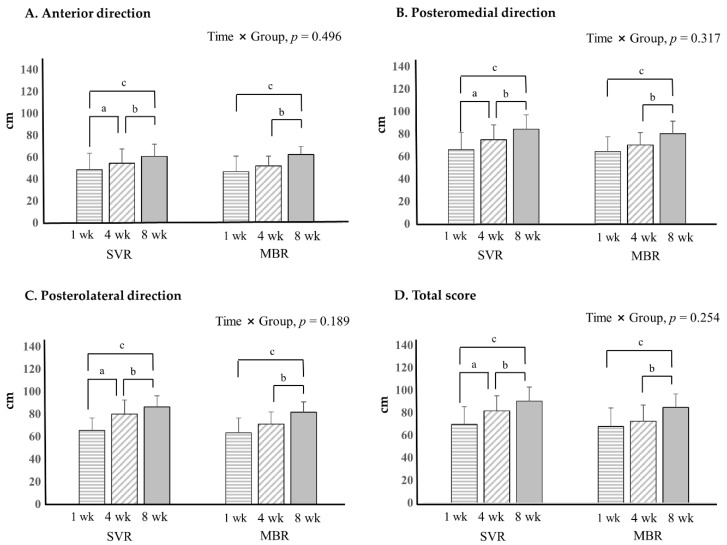
Dynamic balance with Y–balance test. *p* < 0.05; significance, a = 1 week versus 4 weeks; b = 4 weeks versus 8 weeks; c = 1 week versus 8 weeks. Abbreviations: SVR, supervised rehabilitation; MBR, mobile-based rehabilitation, wk; week.

The SVR and MBR groups exhibited significant improvement in the anterior, posteromedial, and posterolateral dynamic balance tests (Figure 5). The SVR group exhibited a significant improvement in all directions at 4 weeks, but not at 1 week, whereas there was no significant improvement in MBR at 4 weeks.

### 3.4. Foot and Ankle Outcome Score Questionnaire

Subjective evaluation of the ankle was performed using the FAOS, and there was a significant improvement in both the SVR and MBR groups. There was a significant improvement in the SVR group at week 4 compared to week 1, indicating that the SVR group recovered rapidly in terms of satisfaction. The interactions according to time and group were significant (Table 3).

## 4. Discussion

The purpose of this study was to analyze the effect of using SVR at a training center and MBR at home or in teams for ankle sprains, which are common in adolescent football players.

The main results of this study indicated that both supervised and at-home rehabilitation training were effective in improving muscle strength, dynamic balance, and subjective satisfaction. A previous study revealed that supervised exercise was more effective in reducing pain and subjective instability, as well as increasing ankle strength and joint position sense than home-based exercise in patients with ankle sprains [18]. Additionally, Cleland et al. [33] in a study conducted for 6 months reported that after an ankle sprain, the group that received manual therapy and training from an expert at the center achieved a superior effect on the Foot and Ankle Ability Measure and Lower Extremity Functional Scale compared to that achieved by home–based training. In one study, the relapse rate was slightly lower at 22% in the home training group compared to 33% in the control group [17].

In the present study, positive results were obtained not only in SVR but also in the MBR group. Experts involved in supervision provided immediate feedback to participants when center-based training was more effective; therefore, training with the correct posture would improve muscle strength and neuromuscular control [34]. Meanwhile, since the researchers provided education, counseling, and feedback to the MBR group through mobile devices, they would have been able to obtain the same positive results as the SVR group. Previous studies have reported that training using a mobile device is a positive experience. Argent et al. [35] reported that exercise non-adherence at home was estimated to be 50%, and that remote coaching using a mobile phone or tablet increases adherence and increases the effectiveness of self-monitoring and education. In a mobile intervention study in patients with chronic ankle instability, eight weeks of home-based balance training significantly improved anterior, posterior, and vertical postural instability, as well as all subjective stability scores compared to those of the control group [36].

However, the natural healing that affects these improvements should not be overlooked. The pain associated with lateral ankle sprains tends to improve over time. D’Hooghe et al. [37] suggested that the healing process after an ankle injury involves an inflammatory phase (2–3 weeks), a proliferative phase (6–12 weeks), and a remodeling phase (up to 1 year). It is therefore possible that there was a reduction in pain during the inflammatory phase and the function of the ankle was restored.

In general, ligament healing can occur from 6 weeks to 3 months following ligament injury due to ankle sprain, objective laxity, and subjective ankle instability can last up to 1 year [38]. In another study, 31% of patients were positive in the anterior pull test after 6 months, and 7–42% complained of instability even after 1 year [39]. Nevertheless, most high school players reported that they returned to sports less than a week following the ankle sprain, which is considerably shorter than the physiological ligament healing time [13]. Based on this information, it appears that many athletes do not receive systematic injury management and return to sports immediately after the inflammatory period.

The reason that players return without adequate recovery and rehabilitation, and the barrier to participating in rehabilitation may be due to psychological anxiety associated with leaving the team or being injured [40]. Although there is a high recurrence rate, injuries that are serious enough to require surgery are very rare. In the study by Guillodo et al. [41], only one of 90 ankle sprains required surgery.

In particular, high school players experience various difficulties that prevent them from going to the center. If they had to visit a center that was a long distance away, there were additional barriers in terms of distance and transportation, particularly in the case of high school students without driving licenses. In addition, the recent closure of sports facilities and social distancing requirements due to coronavirus disease 2019 made visiting the center more difficult [42].

Our rehabilitation program included strength training and measured inversion and eversion strength. A previous study reported that there was no evidence that voluntary muscle strength predicts ankle sprains. Therefore, the role of high muscle strength in preventing ankle sprains remains unknown [43]. A study analyzing the characteristics of ankle sprain patients reported that the loss of dynamic balance ability was greater than the loss of muscle strength [44].

In contrast to muscle strength, postural sway and possibly proprioception related to muscle control are predictors of ankle sprains [43]. In addition, the relative risk that could be prevented by balance training was lowered from 0.15 to 0.40 [45], and it is unlikely that a preventive function could be achieved from strength training alone [46,47]. In a study comparing both strength and balance programs in ankle patients, both dynamic balance and muscle strength were improved in the balance-based training group, but only the muscle strength improved in the strength-based training group [20]. Therefore, emphasizing balance training and increasing the weight of training will be important guidelines for ankle sprain rehabilitation and prevention.

The strength of this study is that it tested the effectiveness of training conducted at home using widely available mobile devices. Therefore, in the field of practice, a program of strength and balance training will be beneficial to patients with ankle sprains based on this study, and 8 weeks of training is required to for it to be effective.

A limitation of this study is that the sample size was relatively small, even though ankle sprains are common. A longitudinal study with a large cohort is required to demonstrate the effectiveness of rehabilitation training in preventing new and recurrent injuries. Further, the training group assignment was not random, but reflected the individual preferences of the participants. We had to consider personal circumstances, such as distance, cost, and time to the center. Further research is necessary because the activities required for each field position vary, and the female football population has also increased recently. With the development of the game industry, digitization, the widespread use of mobile devices and tablets, and the development of various apps for content, it is highly likely that exercise at home will gradually expand [16,48]. Therefore, it would be very meaningful to conduct a study to enhance compliance, participation, and quality with greater effect.

## 5. Conclusions

Training conducted for 8 weeks improved the strength, dynamic balance, and subjective satisfaction of the SVR and MBR groups. The SVR group exhibited rapid improvement in dynamic balance, eversion and dorsiflexion strength at week 4 and 8, whereas MBR did not improve at week 4, but eventually showed improvement at week 8. Therefore, MBR should be an appropriate alternative for recovery in patients with recurrent ankle sprains in an environment where it is difficult to perform SVR.

## Figures and Tables

**Table 1:** Outline of rehabilitation program.

Week	Strength Training with Band and Weight	Balance Training with BOSU	Duration and No. of Sessions
1–2	Band color: red Ankle inversion, eversion Squat	BOSU: hard surface One or two leg stand	15 min 2 sessions per day
3–4	Band color: green Ankle inversion, eversion, plantarflexion Squat, heel raise	BOSU: hard surface One leg squat	15 min 2 sessions per day
5–6	Band color: blue Ankle inversion, eversion, plantarflexion, dorsiflexion Squat, heel raise, toe up Hip abduction-adduction	BOSU: soft surface One leg squat and twist	30 min 1 time per day
7–8	Band color: gray Ankle inversion, eversion, plantarflexion, dorsiflexion Squat, heel raise, toe up Hip abduction, adduction, flexion, extension	BOSU: soft surface One leg squat and twist Jump and change direction	30 min 1 time per day

**Table 2:** General characteristics of participants.

Variables	SVR (*n* = 30)	MBR (*n* = 30)	*t* or χ^2^	*p*-Value	E.S
Age, years	16.3 ± 1.2	16.0 ± 1.3	0.866	0.390	0.239
Height, cm	174.9 ± 6.7	173.9 ± 5.0	0.63.5	0.528	0.169
Weight, kg	64.8 ± 9.6	65.3 ± 5.9	−0.248	0.805	0.006
BMI, kg/m^2^	21.1 ± 1.9	21.5 ± 1.2	−1.149	0.255	0.251
Lateral ankle sprain in the last month					
1–2 times	19 (63.3%)	16 (53.3%)	1.257	0.533	0.145
3–4 times	8 (26.7%)	12 (40.0%)			
>5 times	3 (10.0%)	2 (6.7%)			
Playing position, *n* (%)					
Goal keeper	3 (10.3%)	2 (6.7%)	2.912	0.405	0.220
Defender	7 (23.3%)	9 (30.0%)			
Mid–fielder	17 (56.7%)	12 (40.0%)			
Forward	3 (10.3%)	7 (23.3%)			
Injury side, *n* (%)					
Right	16 (53.3%)	17 (56.7%)	0.067	0.795	0.034
Left	14 (46.7%)	13 (43.3%)			

Abbreviations: BMI, body mass index; SVR, supervised rehabilitation; MBR, mobile-based rehabilitation; E.S; effect size.

**Table 3:** Foot and Ankle Outcome Score.

Subject Scoring	Week	SVR (*n* = 30)	MBR (*n* = 30)	*t*	*p*-Value	Time × Group, *p*	E.S
Foot and ankle outcome score	1	72.0 ± 7.3	72.8 ± 7.2	−0.435	0.655	0.009	0.297
	4	84.0 ± 7.2 ^a^	77.8 ± 7.6	3.616	<0.001		
	8	92.9 ± 4.5 ^b,c^	90.0 ± 4.7 ^b,c^	2.346	0.022		
	*p*-value	<0.001	<0.001				

*p* < 0.05; significance ^a^, 1 week versus 4 weeks; ^b^, 4 weeks versus 8 weeks; ^c^, 1 week versus 8 weeks. Abbreviations: SVR, supervised rehabilitation; MBR, mobile–based rehabilitation; E.S; effect size.

## Data Availability

The data are not publicly available because of privacy or ethics.
